# A comparative study on the distinct staygreen characteristics of two ecological types of *Leymus chinensis* (Poaceae)

**DOI:** 10.3389/fpls.2025.1707645

**Published:** 2025-11-13

**Authors:** Gaowa Naren, Baozhu Dong, Haoyang Yu, Lijuan Ma, Xu Yang, Riqin Hao, Xinxia Wang, Huihui Shi, Lingang Zhang

**Affiliations:** 1 School of Life Sciences, Inner Mongolia University/Key Laboratory of Herbage and Endemic Crop Biotechnology, Hohhot, China; 2 College of Grassland, Resources and Environment, Inner Mongolia Agricultural University, Hohhot, China

**Keywords:** chlorophyll degradation, gray green, *Leymus chinensis*, NOL, NYC1, staygreen, yellow green

## Abstract

The stay-green phenotype is a valuable trait positively correlating with both forage quality and biomass production in perennial grasses. To date, with the exception of *Festuca pratensis*, no naturally occurring stay-green species have been reported among forage grasses. *Leymus chinensis* is an important forage species that presents two phenotypes: gray–green (GG) leaves and yellow–green (YG) leaves. In this study, we discovered that GG *L. chinensis* exhibited functional stay-green characteristics: both chlorophyll and photosynthesis were maintained at higher levels after 6 days of dark treatment, which is significantly different from that of the YG ecotype. Along with higher level of chlorophyll, GG also maintained high concentrations protein nutrient in darkness. In the case of YG, darkness significantly increased the expressions of *NYC1* and *NOL*, which are responsible for initiating the chlorophyll degradation. However, the dark treatment did not alter the expression of *NOL* in the GG leaves, and the induction of *NYC1* expression by darkness in GG was also significantly weaker than that observed in YG. This discrepancy could account for the slower rate of chlorophyll degradation observed in GG under dark conditions than in YG.

## Introduction

The stay-green phenotype has a very valuable characteristic for perennial grasses that are extensively cultivated for forage purposes. This trait is positively correlated with both forage quality and biomass production, which are closely related to leaf greenness (Thomas et al., 1997; 1999; Xu et al., 2019; Duan et al., 2025). However, leaf senescence is a natural process that is associated with the aging of leaves and can be induced or accelerated by abiotic stressors, which can adversely affect plant growth and productivity (Barken et al., 1997; Zhou et al., 2011). Understanding the molecular mechanisms that regulate leaf senescence is of great significance for perennial forage grasses harvested for their green leaves with high nutritional value.

In recent decades, along with an increasing number of key enzymes involved in chlorophyll metabolism, the metabolic pathways of chlorophyll in plants have been established accordingly. The conversion of chlorophyll *b* (Chl *b*) to chlorophyll *a* (Chl a) is regarded as the initial step in chlorophyll degradation, which is activated by two isozymes of Chl *b* reductase, Non-Yellow Coloring 1 (NYC1) and NYC1-like (NOL) (Kusaba et al., 2007; Horie et al., 2009; Sato et al., 2009). Both of these enzymes are responsible for degrading Chl *b* to 7-hydroxymethyl Chl *a* (HMChl *a*) in the chlorophyll cycle. 7-hydroxymethyl chlorophyll *a* reductase (HCAR) has been identified as the enzyme responsible for catalyzing the second step of the reduction of Chl *b* to Chl *a*: the conversion from HMChl *a* to Chl *a* (Meguro et al., 2011). The porphyrin macrocycle of Pheide a undergoes cleavage to produce the red-colored catabolite (RCC) in the subsequent degradation step, which is catalyzed by pheophorbide an oxygenase (PAO) (Pruzinská et al., 2003). As a PAO-bound intermediate, RCC is promptly reduced at the C15/C16 double bond by red chlorophyll catabolite reductase (RCCR), resulting in the formation of colorless primary fluorescent chlorophyll catabolites (pFCCs) (Hörtensteiner et al., 2000; Wüthrich et al., 2000). Afterward, both pFCCs and hydroxy-pFCCs are exported from the chloroplasts and subsequently undergo further modifications in the cytoplasm (Christ et al., 2014). The absence of genes encoding the aforementioned key enzymes always inhibits chlorophyll degradation, resulting in a stay-green phenotype in plants.

*Leymus chinensis* (2n = 4× = 28; NsNsXmXm) is a crucial species in the Eurasian steppe and is characterized by its wide distribution and utilization for grazing. This species is highly valuable for grassland restoration because of its robust rhizome network and exceptional ability to grow in barren soils and tolerate stresses such as salt, alkali, freezing temperatures and drought (Sun and Hong, 2013; Ma et al., 2014; Li et al., 2024). In addition to its ecological advantages, *L. chinensis* is recognized as a valuable pasture grass because of its abundant foliage, palatability, and high nutritional content and is commonly known as sheep grass (Chen et al., 2019). Under natural conditions, *L. chinensis* has evolved two easily distinguishable phenotypes, those with gray–green (GG) leaves and yellow–green (YG) leaves. These two ecotypes exhibit a mosaic distribution and coexist within the same geographic, climatic, and soil environments in the wild (Zhou et al., 2012; 2014; Sun et al., 2022). Research on the divergent adaptations of *L. chinensis* has yielded certain results in terms of genetic characteristics (Wang et al., 2005; Gong et al., 2007; Yuan et al., 2016), seed germination (Ma et al., 2021), stress tolerance (Zhou et al., 2013; Lu et al., 2020) and population dynamics (Yang and Zhang, 2006; Zhou et al., 2014). Collectively, these studies have demonstrated from multiple perspectives that compared with the YG ecotype, the GG-type *L. chinensis* has greater environmental adaptability.

Although some artificially stay-green plants have been created through biotechnology along with studies of chlorophyll degradation mechanisms, naturally occurring stay-green species remain scarce. With the exception of *Festuca pratensis* (Thomas and Stoddart, 1975; Hauck et al., 1997), no natural stay-green species have been reported among forage grasses. In this study, we discovered that compared with YG, GG *L. chinensis* exhibited a natural stay-green trait and that its chlorophyll content was relatively high level after 6 days of dark treatment, which was significantly different than that of YG.

## Materials and methods

### Plant materials, growing conditions and treatments

The seeds of the GG and YG ecotypes of *Leymuch chinensis* were collected in August from the Chilechuan Grassland in Hohhot, Inner Mongolia, China (111°55′ E, 40°54′ N; altitude, 1040 m). In May of the following year, seeds from both ecotypes were sown in soil within a greenhouse under light conditions of ~80 µmol photons m^-^² s^-^¹ and a 12 h light/12 h dark cycle and constant temperature of 23°C were maintained. After a three-week growth period, the majority of seedlings from both the GG and the YG were subsequently transplanted into a field located in Yuquan district of Hohhot city, Inner Mongolia, China (111°42′ E, 40°43′ N; altitude 1035 m), the remaining seedlings of both ecotypes were retained in the greenhouse. The soil type is chestnut soil. The leaves utilized in this study were sourced from plants that had been grown in the field for two years. Five fully expanded second leaves (from the shoot apex) were sampled from five different mature plants of each ecotype. The detached leaves were placed in square Petri dishes, which were then wrapped in aluminum foil to prevent exposure to light, and subsequently placed in a greenhouse maintained at a constant temperature of 23°C. Leaf samples were collected at various time points during the dark treatment at 0, 2, 4, and 6 days. Five biological replicates of the dark treatment were conducted for both ecotypes.

### Determination of chlorophyll and carotenoid contents

Both chlorophyll (Chl) and carotenoids (Cars) were extracted with 80% (v/v) acetone. The Chl *a*, Chl *b* and Car contents were determined by measuring the absorbances at 663 nm, 646 nm and 470 nm, respectively, with a spectrophotometer (DU-800 UV/visible spectrophotometer, China). Quantifications of Chl or Car were performed following the equations of Lichtenhaler (Lichtenthaler, 1987): Chl *a* (µg ml^-1^) = 12.21×A_663_-2.81×A_646_, Chl *b* (µg ml^-1^) = 20.13×A_646_-5.03×A_663_, and total Chl (µg ml^-1^) = Chl *a*+ Chl *b*, Cra (µg ml^-1^) = (1000 × A_470_-3.27 × Chl *a*-104 × Chl *b*)/229.

### Chlorophyll fluorescence measurements

Chlorophyll fluorescence was measured using a MAXI-IMAGING-PAM fluorometer following the manufacturer’s instructions (Walz, Germany). Prior to each measurement, the samples were dark-adapted for 30 min. The minimum fluorescence (*F*_o_) and maximum fluorescence (*F*_m_) yields were measured at a weak beam (0.5 μmol protons·m^− 2^·s^− 1^) and a saturating pulse of light (2700 μmol protons·m^− 2^·s^− 1^ for 0.8 s), respectively. Fluorescence kinetics were induced by activating the light for 5 min; subsequently, the maximum fluorescence under light adaptation (*F*_m′_) and the actual fluorescence (*F*) were measured by applying the saturating pulse every 20 s. All relevant fluorescence parameters, including the maximum quantum yield of PSII (*F*_v_/*F*_m_), effective photochemical quantum yield of PSII [Yield(II)], and nonphotochemical quenching (NPQ), were automatically provided by the instrument.

### Transmission electron microscopy

The *L. chinensis* leaves were cut into 1 mm × 1 mm squares and promptly immersed in an electron microscope fixative solution for fixation. A vacuum pump was then employed to eliminate air until the samples settled to the bottom of the container. The samples were allowed to remain at room temperature for 2 h before being transferred to a refrigerator set at 4°C. Subsequently, through a series of processes, including dehydration, fixation, and embedding, ultrathin sections with thicknesses ranging from 60 nm to 80 nm were prepared using an ultrathin sectioning machine (Leica UC7, Germany). Following double staining with uranium and lead, these sections were examined under a transmission electron microscope (HT7700, Japan) to capture images for further analysis.

### Blue native gel for thylakoid membrane protein analysis

Equal fresh weights of each sample were ground with extraction solution (400 mM sucrose, 10 mM NaCl, 5 mM MgCl_2_, 20 mM Tris, pH 7.8) on ice. First, the mixture was centrifuged at 200×g for 2 min, after which the supernatant was centrifuged again at 4°C and 5,000×g for 10 min. The thylakoid membranes that precipitated at the bottom of the centrifuge tube were slowly resuspended in the extraction solution and stored in liquid nitrogen for the next steps.

The gel was prepared at room temperature using a gradient mixer following the method of Zhang et al (Zhang et al., 2012). The separation gel had a concentration gradient of 5–13.5%, and the concentration of the stacking gel was 4%. The thylakoid membranes were washed with a solution of 25 mM BisTris-HCl and 20% glycerol and then centrifuged at 14,000×g for 5 min. The pellet was suspended in 1% n-dodecyl-b-maltoside (DM), incubated for 1 h on ice, and then centrifuged at 14,000 ×g for 10 min at 4 °C. The supernatant was mixed with a 1/10 volume of Serva G (5% Serva G, 100 mM BisTris-HCl (pH 7.0); 0.5 M 6-aminocaproic acid; and 30% glycerol) thoroughly and then loaded for electrophoresis in an equal volume. The cathode buffer was composed of 50 mM Tricine and 15 mM BisTris (pH 7.0), and the anode buffer was composed of 50 mM BisTris-HCl (pH 7.0). Electrophoresis was carried out at 4 °C. The electrophoresis system was a Hoefer SE250 Mighty Small II Mini Vertical Protein Electrophoresis unit (Hoefer, USA), with a gel thickness of 0.75 mm.

### Soluble protein extraction and SDS–PAGE

Equal fresh weights of each sample were ground with extraction buffer (50 mM Tris-Cl (pH 7.5), 2 mM DTT, 50 mM NaCl, and 5 mM EGTA) supplemented with 1 mM phenylmethanesulfonyl fluoride. The extract was mixed with the same volume of 2×SDS loading buffer (125 mM Tris-Cl (pH 6.8), 2% [w/v] SDS, 5% [v/v] glycerol, 5% [v/v] 2-mercaptoethanol, and 0.05% [w/v] bromophenol blue) and continuously denatured at 95 °C for 5 min. Samples were separated by SDS–PAGE. The resolved gels were stained with Coomassie Brilliant Blue R250.

### Analysis of nutritional composition

*L. chinensis* leaves were dried in an oven at 60 °C for 48 h and subsequently ground to pass through a 1-mm sieve for further nutritional analysis. The crude protein (CP) contents were determined using the Kjeldahl method following the protocol established by the Association of Official Analytical Chemists (AOAC, 2000). CP was calculated as the concentration of Kjeldahl nitrogen multiplied by a factor of 6.25. Crude fiber (CF) analysis was performed according to the Weende method (Pearson, 1996). The neutral detergent fiber (NDF) and acid detergent fiber (ADF) contents were measured in accordance with the National Standards of the People’s Republic of China (GB/T 20806–2006 and NY/T 1459-2007, respectively).

### Analysis of the transcriptome

Total RNA was extracted from each sample using TRIzol^®^ reagent (Invitrogen, USA) following the manufacturer’s protocol. RNA integrity and purity were tested by agarose gel electrophoresis, and the concentrations were measured with a Qubit 2.0 fluorometer (Thermo Fisher Scientific, USA). Once the quality was confirmed, the mRNAs were obtained using polyA tail enrichment of RNAs through oligo(dT) magnetic beads. A Hieff NGS™ MaxUp Dual-mode mRNA Library Prep Kit for Illumina^®^ (Yeasen, China) was used for RNA fragmentation, double-stranded cDNA synthesis, sticky end repair, terminal dA tailing, joint connection, ligation product purification, fragment size sorting and library amplification. After the recovered cDNA was accurately quantified with a Qubit 2.0 fluorometer, Illumina double-ended sequencing was performed on an Illumina HiSeq X Ten system (Illumina, USA).

The sequenced raw data were evaluated using FastQC (version 0.11.2) and trimmed with Trimmomatic (version 0.36). Because there is no reference genome for *L. chinensis*, Trinity (version 2.4.0) software was used to carry out *de novo* assembly of the clean reads into transcript sequences. The resulting spliced transcript was used as a reference sequence for redundant assessment, and the longest transcript in each transcript cluster was treated as a unigene in subsequent analyses. The functional annotation of the compiled transcripts was performed using NCBI Blast+ (version 2.60), which searches against major databases, such as NCBI nucleotide sequences (NT), NCBI nonredundant protein sequences (NR), protein families (PFAM), a manually annotated and reviewed protein sequence database (SwissProt), and euKaryotic Ortholog Groups (KOG). On the basis of the Kyoto Encyclopedia of Genes and Genomes (KEGG) database, the KASS (version 2.1) server was used to provide an extensive overview of the metabolic pathways. To identify genes whose expression significantly differed, we established the following screening criteria: q value < 0.05 and |fold change|>2.0. Three replicates were performed for each treatment.

### RT–PCR

Total RNA was extracted from each sample using TRIzol^®^ reagent (Invitrogen, USA), dissolved in nuclease-free water and treated with DNase I to remove possible DNA contamination. After total RNA was synthesized, the resulting cDNA samples were individually diluted 10× prior to qPCR. RT–PCR was performed with a 2×SG Fast qPCR Master Mix kit (BBI, China) on a StepOne Plus real-time PCR system (ABI, USA). The relative gene expression levels were determined using the comparative 2^-ΔΔCt^ method (Livak and Schmittgen, 2001). The expression level of the actin gene was used as an internal control. The PCR was initiated at 95 °C for 3 min, followed by 45 cycles including 95 °C for 5 s for melt and 60 °C for 30 s for annealing/extension. The error bars indicate the standard errors. The asterisks denote significant differences (One-way ANOVA, * P<0.05, ** P<0.01). The primers used for each gene are listed in **Supplementary Table S1**. Three replicates were performed for each gene.

## Results

### Compared with that in YG leaves, the degradation of chlorophyll in GG leaves occurred at a slower rate during dark induction

**Figure 1:**
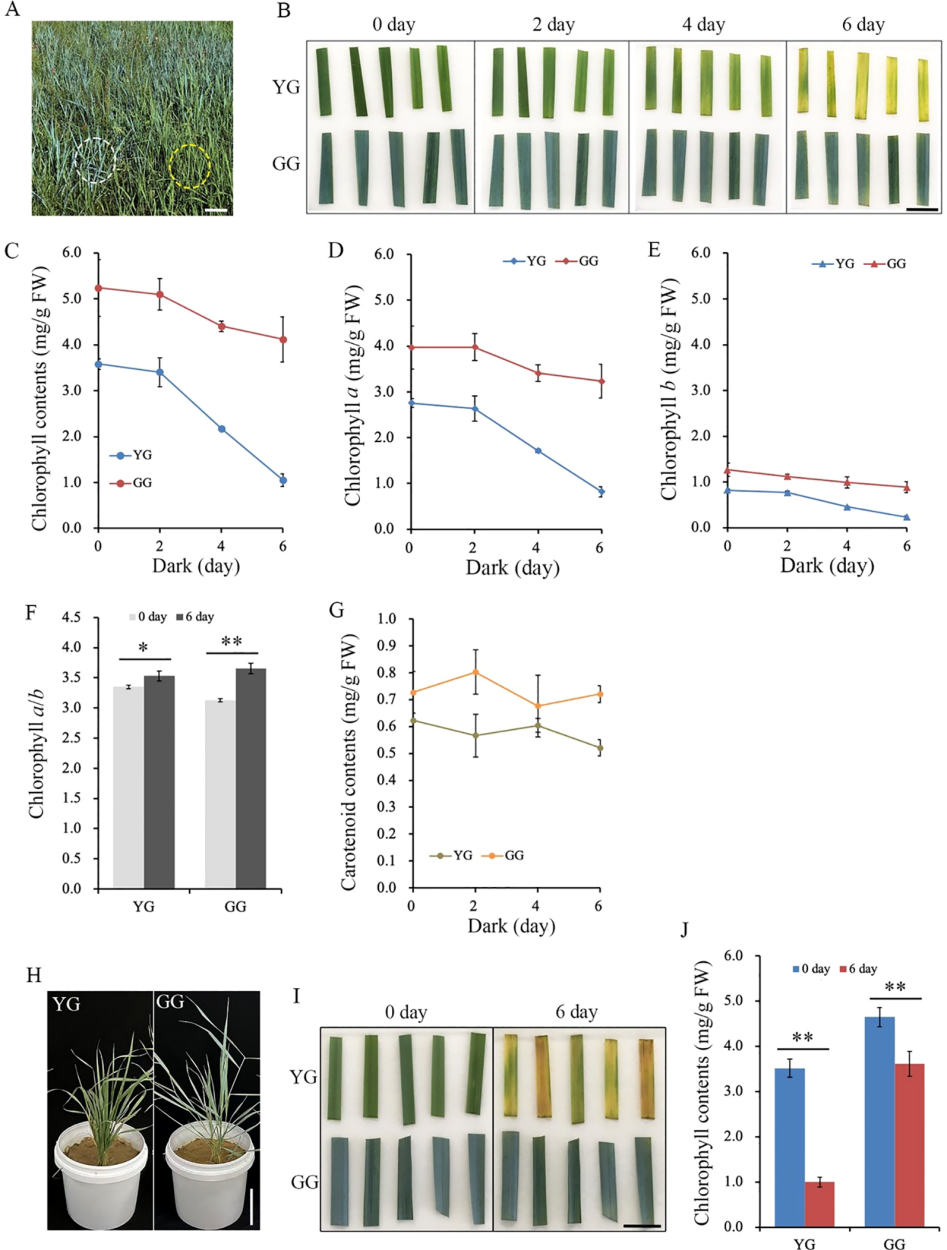
The chlorophyll degradation of YG and GG under dark conditions. **(A)** The phenotypes of YG and GG observed in the field. The yellow dashed circle indicates YG, while the white dashed line marks GG. Bar = 15.0 cm. **(B)** The alterations in leaf color of YG and GG following dark treatment for 0, 2, 4, and 6 days. Bar = 2.0 cm. **(C)** Decrease in leaf chlorophyll content with prolonged duration of dark treatment. The levels of chlorophyll *a***(D)** and *b***(E)** in the leaves of both YG and GG declined with extended dark treatment. **(F)** The ratio of chlorophyll *a* to *b* before and after dark treatment. **(G)** The carotenoid contents of YG and GG under dark conditions. **(H)** The phenotypes of YG and GG cultivated in the greenhouse. Bar = 15.0 cm. **(I)** The leaf color of YG and GG before (0 day) and after dark treatment (6 day). Bar = 2.0 cm. **(J)** Decrease in leaf chlorophyll content of YG and GG before (0 day) and after dark treatment (6 day). YG, yellow–green *Leymus chinensis*. GG, gray–green *Leymus chinensis*. The error bars indicate S.D. Asterisks denote significant differences (One-way ANOVA, * P<0.05, ** P<0.01).

Darkness, as an extreme light condition, is frequently employed to induce rapid and synchronous senescence in detached leaves (Weaver and Amasino, 2001). Mature leaves were harvested from the field-grown YG and GG and subjected to dark treatment (**Figure 1A**). After being incubated in the dark for 6 days, all the YG leaves exhibited a yellow coloration, whereas the GG leaves still remained gray green (**Figure 1B**). The chlorophyll contents in the leaves were measured after 0, 2, 4, and 6 days of dark treatment. Consistent with the observed changes in leaf color shown in the upper panel, chlorophyll degradation occurred significantly faster in YG leaves than in GG leaves (**Figure 1C**). The chlorophyll content in YG *L. chinensis* decreased by 70%, from 3.6 mg/gFW to 1.1 mg/gFW, after the dark treatment for 6 days, whereas that in GG *L. chinensis* decreased by only 20% (5.2 mg/g FW to 4.3 mg/gFW) under the same conditions. Not only did the level of Chl *a* significantly decrease, but the level of Chl *b* markedly decreased in the dark (**Figures 1D, E**). Chl *a*/*b* increased after 6 days of dark treatment compared with the control (0 days of darkness) for both ecotypes (**Figure 1F**). Unlike chlorophyll, only a slight reduction in carotenoid levels was observed in YG, while no significant changes in carotenoid content were detected in GG after the 6-day dark treatment (**Figure 1F**). Similar to the data from *L. chinensis* growing in the field, YG and GG, which were continuously cultivated in the greenhouse, also demonstrated different rates of chlorophyll degradation when subjected to darkness: the reduction of chlorophyll in YG occurred at a significantly faster rate compared to that in GG. (**Figures 1H–J**). The above results suggested that GG *L. chinensis* was capable of sustaining a relatively high chlorophyll content under dark conditions and exhibited a typical stay-green phenotype.

### The higher chlorophyll contents in GG leaves under dark conditions contributed to the maintenance of photosynthetic performance

**Figure 2:**
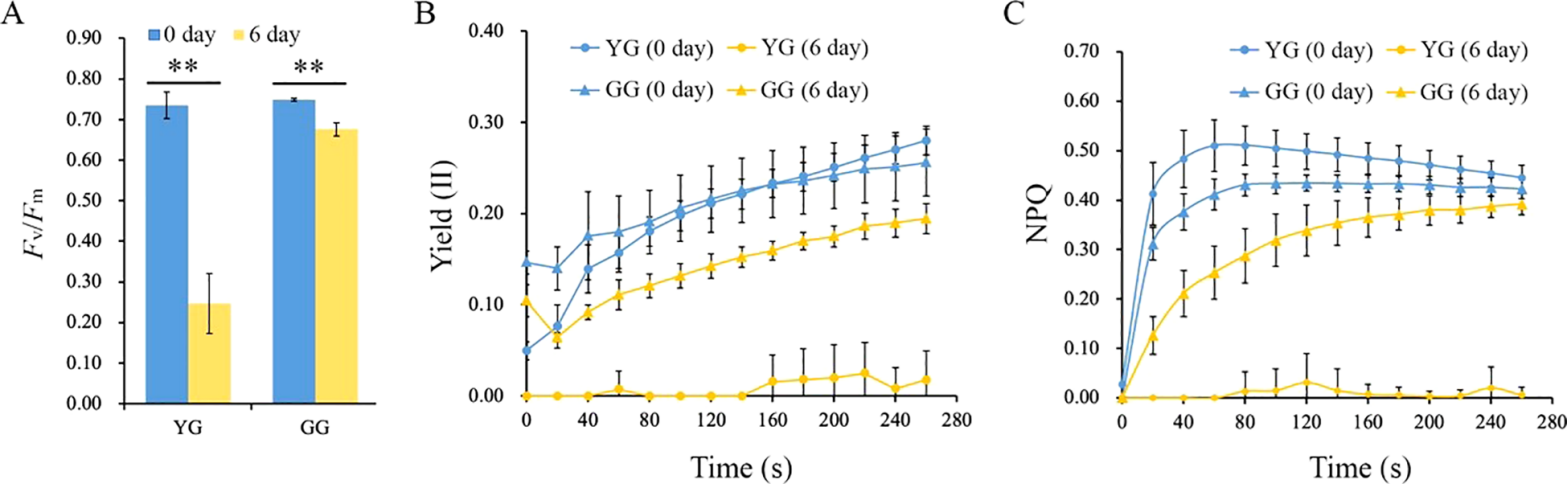
The photosynthetic activities of YG and GG before and after dark treatment. **(A)***F*_v_/*F*_m_ of YG and GG. **(B)** Yield (II) of YG and GG leaves subjected to darkness. **(C)** Variations in NPQ for YG and GG leaves under dark conditions. YG, yellow–green *Leymus chinensis*. GG, gray–green *Leymus chinensis*. Five independent plants from each ecotype were used for photosynthesis analysis. The error bars indicate S.D. Asterisks denote significant differences (One-way ANOVA, ** P<0.01).

Chlorophyll serves as the primary collector of light energy, facilitating the process of photosynthesis within chloroplasts. The depletion of chlorophyll clearly affects the photosynthetic activity of *L. chinensis. F*_v_/*F*_m_, which represents the maximum photochemical quantum yield of photosystem II, decreased for both ecotypes after a 6-day period of darkness. The *F*_v_/*F*_m_ of YG decreased from 0.72 to 0.23, which was notably lower than that of GG, whose value decreased from 0.73 to 0.68. (**Figure 2A**). The Yield(II) represents the actual light energy conversion efficiency of the leaf. After the 6-day dark treatment, the Yield(II) of YG decreased to its lowest level, whereas the Yield(II) of GG remained relatively high, despite it declining relative to that of the control (**Figure 2B**). The higher levels of *F*_v_/*F*_m_ and Yield(II) suggested that GG maintained a high degree of photosynthetic competence even when it was subjected to 6 days of darkness. Consistent with Yield(II), nonphotochemical quenching (NPQ) was significantly reduced in dark-treated YG leaves. In contrast, the NPQ of GG remained high after darkness (**Figure 2C**). Both the photosynthetic capability and the protective mechanisms of photosystem II clearly functioned effectively in GG leaves, whereas this was not the case for YG leaves. These findings indicated that the chlorophyll retained in GG under dark conditions could efficiently sustain its photosynthetic performance.

### Differences in the chloroplast morphology and structure of the two *L. chinensis ecotypes*

**Figure 3:**
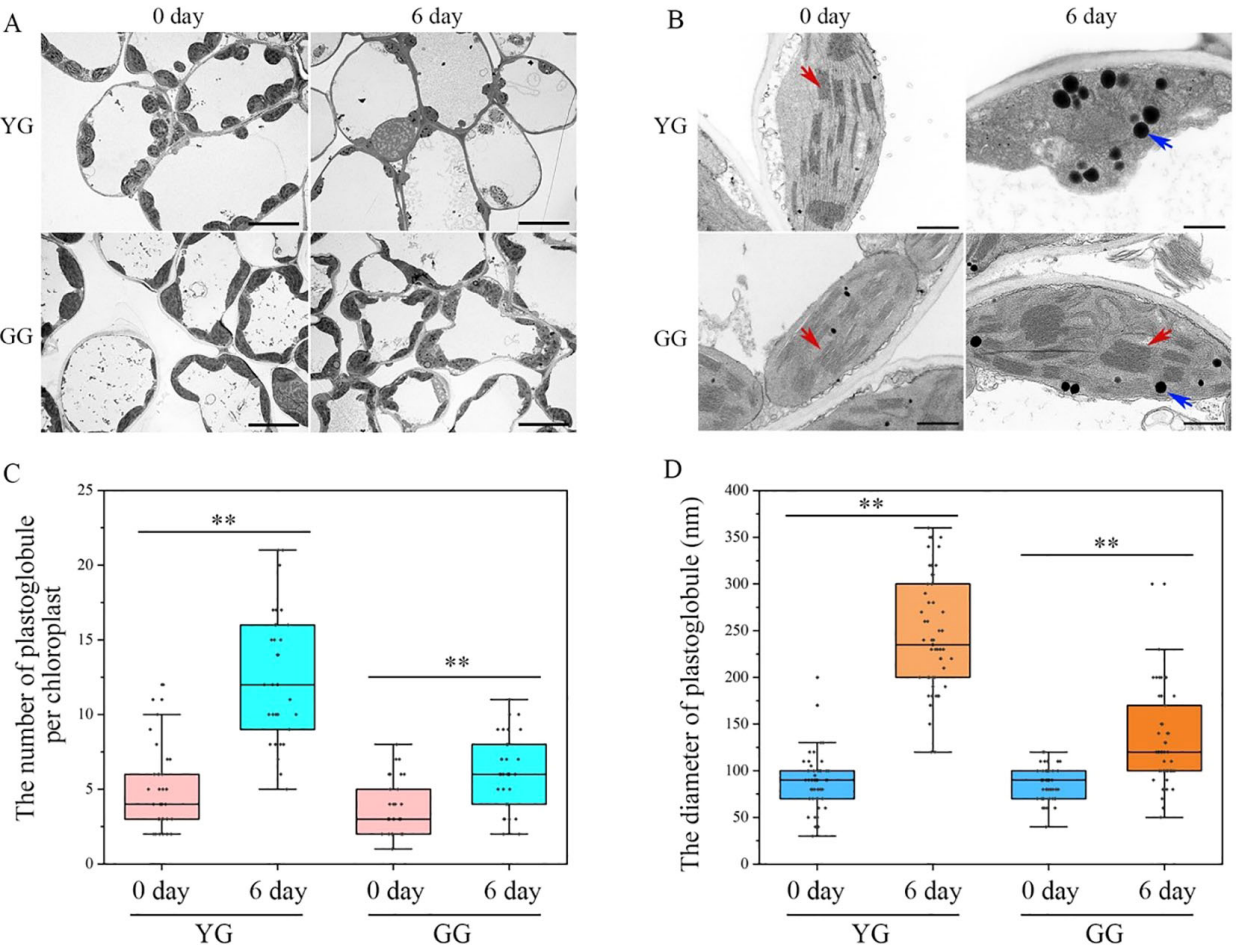
The transmission electron microscopies of chloroplasts in YG and GG. **(A)** The chloroplasts observed in the cells of YG and GG leaves. **(B)** The representative images of chloroplasts from YG and GG. The blue arrowheads indicate globular structures, and the red arrowheads denote thylakoid membranes. Bars=10.0 µm **(A)**; bars=1.0 µm **(B)**. **(C)** The quantitative analysis of plastoglobules within each chloroplast. Thirty chloroplasts from each sample were employed for the quantification of plastoglobules. **(D)** The variation in the size of plastoglobules within chloroplasts. A total of fifty plastoglobules derived from different chloroplasts were analyzed for their diameters. The error bars indicate S.D. Asterisks denote significant differences (One-way ANOVA, ** P<0.01). YG, yellow–green *Leymus chinensis*. GG, gray–green *Leymus chinensis*.

The reduction in chlorophyll in the leaves of plants following dark treatment inevitably affects the morphology and structure of chloroplasts, frequently resulting in chloroplast degradation. Transmission electronic images of the ultrathin sections of leaves from YG and GG are presented in **Figure 3A**. The chloroplasts in both ecotypes exhibited canonical lens-shaped structures positioned at the edges of the cells. After the dark treatment, the morphology of the YG chloroplasts significantly changed, with irregularly shaped chloroplasts replacing the previously lens-like structures. In the case of GG, normal chloroplasts were still observed in proximity to the cell walls, similar to those found in the control group. After a 6-day incubation in darkness, the thylakoid systems within the YG chloroplasts were nearly completely absent, with numerous plastoglobules occupying the organelle instead. However, both grana and lamellar thylakoid membranes can be preserved and clearly distinguished within the GG chloroplasts (**Figure 3B**). Statistical analysis revealed that dark treatment not only significantly enhanced the quantity of plastoglobules within each chloroplast but also markedly increased their size. The increase in both the quantity and size of plastoglobules in YG was clearly greater than that observed in GG under same conditions (**Figures 3C, D**). The extent of chloroplast degradation is directly proportional to the production of plastoglobules. The smaller number of plastoglobules observed in GG following dark induction suggested that the rate of chloroplast degradation in this ecotype was significantly lower than that in YG.

### Nutrient compositions of leaves from YG and GG subjected to dark treatment

**Figure 4:**
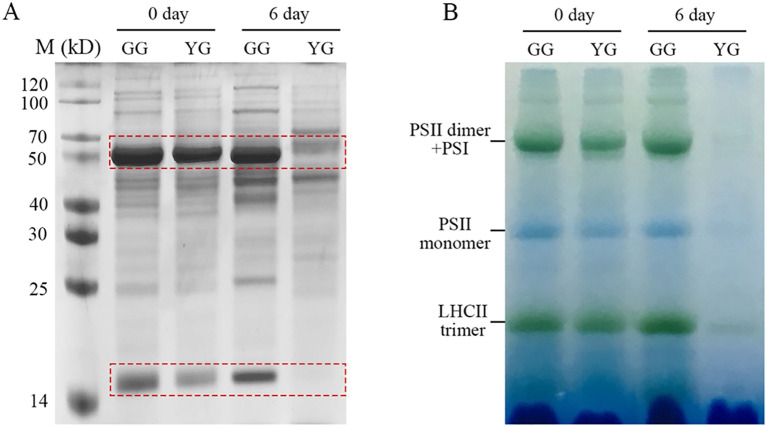
Soluble and thylakoid membrane proteins of GG and YG leaves in darkness. **(A)** Separation of soluble proteins using SDS–PAGE. The dashed rectangles mark the large and small subunits of Rubisco, respectively. **(B)** Characterization of thylakoid membrane proteins from GG and YG with blue-native PAGE. The samples were loaded on the base of equal fresh weight of GG and YG leaves for both gel analysis. YG, yellow–green *Leymus chinensis*. GG, gray–green *Leymus chinensis*.

As a valuable forage, the protein content of *L. chinensis* is a significant nutritional trait. Ribulose-1,5-bisphosphate carboxylase/oxygenase (Rubisco) and Light-harvesting complex II (LHCII) are the most abundant soluble and membrane proteins, respectively, inside chloroplasts. It is intriguing to investigate whether stay-green GG can also maintain protein levels following dark treatment. First, total soluble proteins were extracted from the leaves and separated using SDS–PAGE. Both the larger and the smaller subunits were resolved at the upper (~50 kD) and lower (~15 kD) regions of the SDS–PAGE gel, respectively (**Figure 4A**). In the case of dark-treated YG, both Rubisco subunits were completely lost. In contrast, GG maintained high levels of these proteins even after six days of darkness. Second, a blue native gel was used to separate the protein complexes present in the thylakoid membrane. The protein complexes associated with the YG thylakoid membrane were completely lost following a 6-day period of dark stress. However, the majority of membrane proteins, including Photosystem I (PSI), Photosystem II (PSII), and LHCII complexes, remained present in GG leaves (**Figure 4B**). In summary, both soluble and membrane proteins markedly decreased in YG leaves, whereas both types of proteins were maintained at relatively high levels in GG leaves.

In addition to the direct analysis of proteins through gel electrophoresis, crude protein is another common parameter that is used to describe the nutritional value of forage. Similar to the results obtained from the gel analysis, compared with the control leaves, the dark-treated YG leaves presented a significant reduction in crude protein levels. However, darkness did not induce a loss of crude protein in the GG samples (**Table 1**). In addition to proteins, crude fiber (CF), acid detergent fiber (ADF), and neutral detergent fiber (NDF) are typically used to assess the nutritional quality of grass. The three parameters increased following a period of darkness for both types of sheep grasses (**Table 1**).

**Table 1:** The alterations in nutrient composition before and after darkness.

Ecotype	YG		GG	
Dark (day)	0	6	0	6
Crude Fiber (%)	30.19 ± 0.20	31.60 ± 0.11^**^	28.03 ± 0.11	31.14 ± 0.17^**^
Crude Protein (%)	13.36 ± 0.15	11.39 ± 0.26^**^	12.43 ± 0.49	12.72 ± 0.11
Acid Detergent Fiber (ADF) (%)	24.59 ± 0.11	26.45 ± 0.16^**^	23.38 ± 0.12	32.53 ± 0.15^**^
Neutral Detergent Fiber (NDF) (%)	55.72 ± 0.12	56.46 ± 0.13^**^	57.60 ± 0.20	63.34 ± 0.17^**^

** indicates significant differences at 0.01 levels. Data represent mean ± S.D.

### Analysis of gene expression variations in GG and YG under dark induction

**Figure 5:**
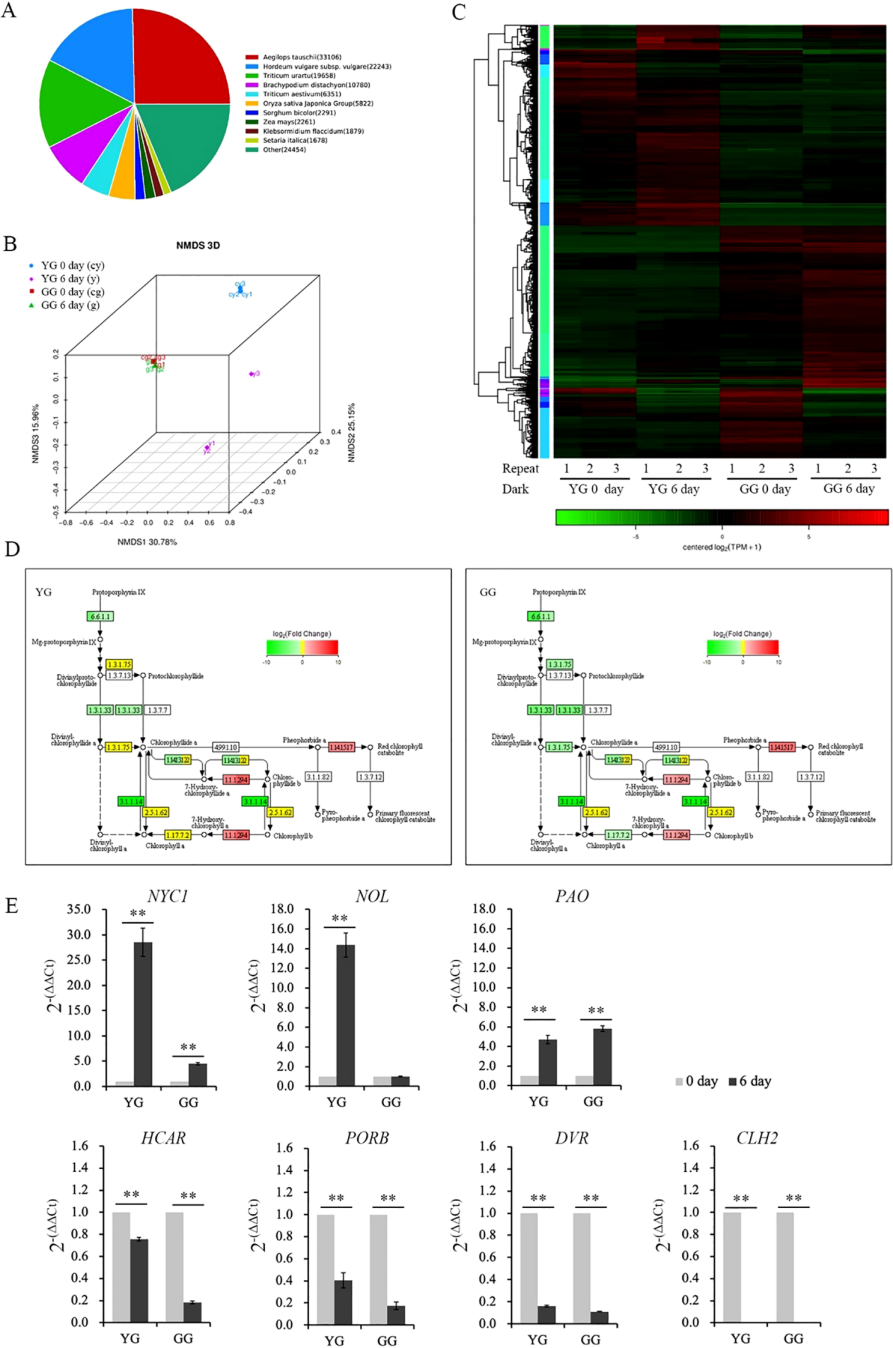
Transcription of genes involved in chlorophyll metabolism in YG and GG under dark conditions. **(A)** Similar species of *Leymus chinensis* identified through transcriptome analysis. **(B)** NMDS analysis comparing different groups of *Leymus chinensis* samples. **(C)** Heatmap illustrating the transcriptional activity of YG and GG before and after dark treatment. **(D)** Partial pathway maps of chlorophyll metabolism derived from KEGG analysis. The different colors of the rectangular boxes indicate variations in transcription: red signifies increased transcription, green denotes decreased expression, yellow indicates no significant changes in transcription level, and white suggests that no expression has been detected. The numbers inside the boxes indicate the enzyme E.C. number. The black lines with arrows denote metabolic pathways, whereas the dashed lines represent the proposed pathways. **(E)** Transcription activity induced by darkness, as determined through real-time PCR analysis. Five independent plants from each ecotype were used for real-time PCR analysis. The error bars indicate S.D. Asterisks denote significant differences (One-way ANOVA, ** P<0.01). YG, yellow–green *Leymus chinensis*. GG, gray–green *Leymus chinensis*.

The transcriptome was used to investigate the gene expressions that regulate chlorophyll degradation in these two *L. chinensis* ecotypes. A similarity comparison of gene sequences indicated that *L. chinensis* is more closely related to *Aegilops tauschii* (**Figure 5A**). NMS 3D analysis demonstrated that the repeat samples were consistent and uniform (**Figure 5B**). The cluster heatmap illustrated the expression levels of the differentially expressed genes in the two ecotypes of *L. chinesis* following dark induction (**Figure 5C**). Furthermore, KEGG analysis of chlorophyll metabolism based on the transcriptomic data revealed that darkness increased (indicated by the red box) the expressions of genes associated with chlorophyll degradation. Concurrently, darkness also suppressed (as shown in the green box) the expressions of genes involved in chlorophyll production (**Figure 5D**; **Supplementary Table S2**). Real-time PCR was conducted to quantitatively validate the expressions of the aforementioned genes. In accordance with the results of the transcriptome analysis, the expression levels of *NYC1*, *NOL*, and *PAO*, genes involved in chlorophyll degradation, increased. Conversely, genes associated with chlorophyll production were downregulated (**Figure 5E**). *NYC1* and *NOL* are responsible for initiating the degradation of chlorophyll by converting Chl *b* to Chl *a*. In the case of YG, darkness significantly increased the expressions of both genes. However, in GG leaves, the dark treatment did not alter the expression of *NOL*. Although the *NYC1* expression in GG increased markedly following dark induction, this increase was considerably lower than that observed in YG (**Figure 5E**). The increase in *PAO* expression was comparable between YG and GG (**Figure 5E**). The increased gene expressions of *NYC1* and *NOL* were significantly lower in GG than in YG. This discrepancy could account for the slower rate of chlorophyll degradation observed in GG under dark conditions than in YG.

## Discussion

Reduced chlorophyll degradation in senescent leaves or during biomass postharvest drying and storage is of particular interest for forage crops because their market value is closely related to the visual appearance of their foliage. The stay-green trait of GG *L. chinensis* clearly indicates that this natural grass plays a valuable role in both marketability and breeding programs. In this study, we obtained the following results: 1) compared with YG *L. chinensis*, GG demonstrated functional stay-green characteristics; 2) the prolonged degradation of chlorophyll in GG could effectively maintain its protein levels under dark conditions; and 3) the expression levels of *NYC1* and *NOL* were relatively low in GG, which could account for the delayed degradation of chlorophyll.

### The delayed degradation of chlorophyll in GG *L. chinensis* was classified as a functional stay-green type

The defining characteristic of stay-green mutants is their ability to retain chlorophyll during periods of darkness or in later developmental stages. However, some kinds of green leaves may have already lost their photosynthetic capabilities. These leaves are merely cosmetic and appear alive. In contrast, there are truly functional leaves that maintain their physiological activity, referred to as functional stay-green species (Thomas and Howarth, 2000), similar to those observed in GG in this study. Both direct observations and chemical assays of chlorophyll contents demonstrated that GG, whether grown in the field or greenhouse, was capable of retaining chlorophyll even after six days of dark treatment, whereas YG could not. Chl *a* and *b* were preserved at relatively high levels in the detached leaves of GG (**Figure 1**). Therefore, the green color of dark-treated GG leaves was attributed to the presence of these two types of chlorophyll molecules.

Chl *a* and *b* are both crucial for maintaining the stability of the LHCII trimer. In the absence of Chl *b*, the dissociation of the trimer into monomers triggers a degradation cascade within photosystems (Hörtensteiner and Kräutler, 2011; Kouřil et al., 2012). Notably, LHCII remains intact even after senescence in mutants lacking *NYC1* (Kusaba et al., 2007) or *NOL* (Sato et al., 2009), indicating that LHC degradation is regulated by Chl *b* reductase. Moreover, the progressive breakdown of Chl *a* destabilizes PSII complexes, leading to the disassembly of the protein-pigment assembly necessary for photochemical energy conversion (Krieger-Liszkay et al., 2008). Clearly, the higher levels of Chl *a* and *b* retained in dark-treated GG leaves contributed to sustaining the stability of LHCII trimers as well as PSI and PSII supercomplexes (**Figure 4B**). Consistently, the majority of the thylakoid system was maintained in GG leaves, whereas numerous plastoglobules, which have traditionally been regarded as products of lipid destabilization (Van Wijk and Kessler, 2017), were observed in YG chloroplasts after dark treatment (**Figures 3B, C**). Consequently, the GG leaves retained their photosynthetic activity, as evidenced by the measurements of *F*_v_/*F*_m_, Yield (II), and NPQ under darkness (**Figure 2**). These findings indicated that the green GG leaves retain their photosynthetic activity rather than merely exhibiting cosmetic greening.

### The stay-green trait of GG contributed to preserving its protein nutritional value in the absence of light

Maintaining the chlorophyll content in plant leaves not only enhances their visual appeal but also significantly contributes to their nutritional value as forage for livestock. Rubisco, a key component of all green leaves, constitutes up to 50% of soluble leaf protein and provides numerous advantageous functional characteristics, including an essential amino acid profile, reduced allergenicity, improved gelation, foaming, emulsification capabilities, and enhanced textural properties (Udenigwe et al., 2017). Following the darkness treatment, both the large (~55.0 kDa) and small (~12.5 kDa) subunits of YG were undetectable on the gel, whereas the reductions in both subunits in GG leaves were less pronounced after incubation in the dark for 6 days (**Figure 3A**). These nondegradable Rubisco proteins render GG more nutritious than YG when subjected to dark-induced senescence. Furthermore, another key nutrient parameter associated with protein, crude protein, is routinely assessed to evaluate forage nutrition. In line with the changes observed in Rubisco levels, the crude protein content of YG decreased significantly, whereas it remained relatively stable in GG (**Table 1**). Consequently, both protein parameters consistently indicated that stay-green GG could maintain its protein levels alongside chlorophyll retention under senescence conditions. A positive correlation between protein levels and chlorophyll content has also been observed in stay-green alfalfa. Transgenic alfalfa plants in which the *SGR* gene was silenced through RNA interference (RNAi) maintained more than 50% of their chlorophyll content during senescence, along with a higher crude protein concentration (Zhou et al., 2011). In addition to crude protein, the other nutrient parameters, such as CF, NDF, and ADF, significantly increased in both ecotypes following the darkness treatment (**Table 1**). This observation suggested that compared with other cellular components, cell wall degradation occurred at a slower rate. In summary, in addition to higher levels of chlorophyll, GG also maintained a high concentration of nutrients in terms of proteins in the dark.

### Weak activation of *NYC1* and *NOL* by darkness was responsible for slower chlorophyll degradation in GG leaves

The conversion of chlorophyll to noncolored FCC involves a series of steps that are strictly regulated by various enzymes, such as NYC1, NOL, HARC, and PAO (reviewed by Tanaka and Ito, 2025). During senescence in monocots, such as rice (Sato et al., 2009) and *Zoysia japonica* (Guan et al., 2022), the expressions of both *NYC1* and *NOL* can be activated. Mutants of *nyc1* or *nol* in rice exhibit a stay-green phenotype along with elevated levels of retained Chl *b* and LHCII (Kusaba et al., 2007; Sato et al., 2009). In contrast, the degradation of Chl *b* is not significantly influenced by the loss of *NOL* in Arabidopsis, suggesting that *NYC1* serves as the primary enzyme regulating Chl *b* levels (Horie et al., 2009). The data from the present study clearly indicated that the expressions of these two genes were negatively correlated with the chlorophyll contents in these two ecotypes. Unlike YG, the limited promotion of *NYC1* and *NOL* expressions under darkness may account for the slight reduction in chlorophyll observed in GG. Unlike that in *NYC1* and *NOL*, *PAO* expressions were similarly enhanced in both ecotypes in darkness (**Figures 5D, E**). Previous evidence has shown that *PAO* is also expressed in stay-green Festuca and Lolium (Vicentini et al., 1995; Roca et al., 2004). Taken together with our findings, it can be concluded that compared with PAO, NYC1 and NOL play a more critical role in the different rates of chlorophyll degradation between GG and YG.

As an enzyme involved in the chlorophyll cycle, HCAR has also been suggested to play a role in chlorophyll degradation (Meguro et al., 2011). However, the deletion of *HCAR* did not prevent the reduction in chlorophyll, and leaves detached from *HCAR* knockout mutants still turned yellow in the dark. Furthermore, the natural senescence of this mutant did not differ from that of wild-type plants, indicating that HCAR is not essential for leaf senescence (Hu et al., 2021). In this study, the expression of *HCAR* was inhibited rather than activated under dark conditions. These findings indicated that chlorophyll degradation in these two ecotypes did not require the involvement of this enzyme. In contrast to the expressions of genes involved in chlorophyll degradation, the expressions of genes involved in chlorophyll synthesis, such as *PORB*, *DVR*, and *CLH2*, were significantly suppressed in both YG and GG (**Figures 5D, E**). Chlorophyll synthesis in these two ecotypes is limited in the dark.

Dark stress leads to the overproduction of reactive oxygen species (ROS) in plants, which causes chlorophyll degradation and leaf senescence (Rosenvasser et al., 2006). Exogenous melatonin has been reported to downregulate chlorophyll degradation in perennial ryegrass, which was attributed to its role in regulating ROS scavenging by activating the superoxide dismutase (SOD)-catalase (CAT) enzymatic antioxidant pathway (Zhang et al., 2016). SOD and CAT levels are also significantly greater in GG than in YG under salt stress, which accounts for the greater tolerance to salt stress observed in GG than in YG (Zhou et al., 2017). It is plausible that the slower degradation of chlorophyll in GG may also be related to its antioxidant capacity under dark stress conditions, similar to its behavior under salt stress.

## Data Availability

The raw data of RNA-seq generated for this study have been deposited in the NCBI SPA (PRJNA1346084, http://www.ncbi.nlm.nih.gov/bioproject/1346084).

## Supplementary material

Supplementary Table 1 Primers used in real-time PCR analysis.

Supplementary Table 2 Genes transcription involved in chlorophyll metabolism based on KEGG analysis.
