# Supplementary material for: A comparative study on the distinct staygreen characteristics of two ecological types of Leymus chinensis (Poaceae)
Source: Front Plant Sci. 2025 Nov 13;16:1707645. doi: 10.3389/fpls.2025.1707645 (PMC12659654; doi:10.3389/fpls.2025.1707645)
Supplement: Supplementary Table 1 — Primers used in real-time PCR analysis. [file DataSheet1.docx]

**Supporting information**

**A Comparative Study on the Distinct Staygreen Characteristics of Two Ecological Types of *Leymus chinensis* (Poaceae)**

**This section includes:**

Table S1

Table S2

**Table S1.** The primers used in real-time PCR analysis.

| Gene | Primer |
| --- | --- |
| *Actin* | Forward: 5’-ATTGTGCTCAGTGGTGGGTCA-3’ |
|  | Reverse: 5’-CCAATCCAAACACTGTACTTCCTC-3’ |
| *NYC1* | Forward: 5’-GGGCTGAAAGTCGAACACG-3’ |
|  | Reverse: 5’-CCGCCTGAACTGGAAAGC-3’ |
| *NOL* | Forward: 5’-CTACCACAAAGCAAGCAAAGTT-3’ |
|  | Reverse: 5’-TGTTCCTACGAGCACCAAAAG-3’ |
| *PAO* | Forward: 5’-CGGAAGCCCTAGCCAAGA-3’ |
|  | Reverse: 5’-TGTCGCACCGAACACCAC-3’ |
| *HCAR* | Forward: 5’-ATTGGCTGGCAGGGAAAA-3’ |
|  | Reverse: 5’-AACGGCACTCGTGAAGGAC-3’ |
| *CLH2* | Forward: 5’-TTCTGGAGCACGGTGTTGA-3’ |
|  | Reverse: 5’-GGATTACGGGCATCTGGAC-3’ |
| *DVR* | Forward: 5’-CGAGGAAAAGGCTAACAAGG-3’ |
|  | Reverse: 5’-CAATCCATCGAGCACCCA-3’ |
| *PORB* | Forward: 5’-CTGGCGGAGTCAGGCAAG-3’ |
|  | Reverse: 5’-CCGTCTGCGGTGAAGGAA-3’ |
| *CH1G* | Forward: 5’-GCCTTATCGTCCTATTCCTTCA-3’ |
|  | Reverse: 5’-TTCCTATCCATCCATTCTGCTT-3’ |

**Table S2.** Transcription of genes involved in chlorophyll metabolism based on KEGG analysis.

| Gene | Gene annotation | EC number | Log_2_(Fold Change) | | Transcript ID |
| --- | --- | --- | --- | --- | --- |
|  |  |  | YG | GG |  |
| *NYC1* | Chlorophyll(ide) b reductase NYC1 | 1.1.1.294 | 5.35 | 3.14 | TRINITY_DN94657_c1_gl |
| *NOL* | Chlorophyll(ide) b reductase NOL | 1.1.1.294 | 3.55 | 2.28 | TRINITY_DN97112_c4_g1 |
| *PAO* | Pheophorbide a oxygenase | 1.14.15.17 | 3.52 | 4.45 | TRINITY_DN67207 _cl_gl |
| *DVR* | Divinyl chlorophyllide a 8-vinyl-reductase | 1.3.1.75 | -0.12 | -2.45 | TRINITY_DN89497_c0_g3 |
| *HCAR* | 7-hydroxymethyl chlorophyll a reductase | 1.17.7.2 | -0.17 | -1.17 | TRINITY_DN90404_c1_gl |
| *CH1G* | Chlorophyll/bacteriochlorophyll a synthase | 2.5.1.62 | -0.41 | -0.21 | TRINITY_DN90666_c1_g1 |
| *CHLD* | Magnesium chelatase subunit Ch1D | 6.6.1.1 | -1.09 | -1.89 | TRINITY_DN94029_c0_g3 |
|  |  |  | -1.37 | -2.18 | TRINITY_DN94029_c0_g2 |
| *CHLI* | Magnesium-chelatase subunit Chl I | 6.6.1.1 | -1.76 | -6.62 | TRINITY_DN87368_cl_gl |
| *PORB* | Protochlorophyllide reductase B | 1.3.1.33 | -1.93 | -4.37 | TRINITY_DN77458_c0_gl |
|  |  |  | -2.43 | -4.14 | TRINITY_DN64285_cl_g2 |
| *CAO* | Chlorophyllide a oxygenase | 1.14.13.122 | -2.65 | -3.00 | TRINITY_DN76156_c0_gl |
|  |  |  | -2.96 | -3.11 | TRINITY_DN94350_cl_g2 |
|  |  |  | -0.47 | 0.44 | TRINITY_DN94350_c1_g1 |
| *CHLH* | Magnesium chelatase subunit Ch1H | 6.6.1.1 | -4.60 | -6.77 | TRINITY_DN97426_c5_gl |
| *CLH2* | Chlorophyllase | 3.1.1.14 | -10.98 | -16.92 | TRINITY_DN71608_c2_gl |
